# Detection of a Multiple Circulation Event of Dengue Virus 2 Strains in the Northern Region of Brazil

**DOI:** 10.3390/tropicalmed9010017

**Published:** 2024-01-09

**Authors:** Murilo Tavares Amorim, Felipe Gomes Naveca, Leonardo Henrique Almeida Hernández, Thito Yan Bezerra da Paz, Cintia Cryslaine da Silva de Oliveira, Alessandra da Conceição Miranda Santos, Alice Louize Nunes Queiroz, Ana Lucia Monteiro Wanzeller, Eliana Vieira Pinto da Silva, Fábio Silva da Silva, Sandro Patroca da Silva, Bruno Tardelli Diniz Nunes, Ana Cecília Ribeiro Cruz

**Affiliations:** 1 Institute of Biological Sciences, Federal University of Pará, Belém 66075-110, Brazil; cintiacryslaine@gmail.com; 2 Department of Arbovirology and Hemorrhagic Fevers, Evandro Chagas Institute, Health and Environment Surveillance Secretariat, Ministry of Health, Ananindeua 67030-000, Brazil; leohenrique96@hotmail.com (L.H.A.H.); alessandrasantos@iec.gov.br (A.d.C.M.S.); alicequeiroz@iec.gov.br (A.L.N.Q.); anawanzeller@iec.gov.br (A.L.M.W.); elianapinto@iec.gov.br (E.V.P.d.S.); spatroca@gmail.com (S.P.d.S.); brunonunes@iec.gov.br (B.T.D.N.); 3 Laboratory of Infectious Diseases Ecology in Amazon, Leonidas and Maria Deane Institute, Fiocruz, Manaus 69057-070, Brazil; felipe.naveca@fiocruz.br; 4 Arbovirus and Hemorrhagic Virus Laboratory, Oswaldo Cruz Institute, Fiocruz, Rio de Janeiro 21040-900, Brazil

**Keywords:** arboviruses, dengue virus serotype 2, cosmopolitan genotype, Asian-American genotype

## Abstract

Dengue virus serotype 2 (DENV-2) is responsible for dengue epidemics on a global scale and is associated with severe cases of the disease. This study conducted a phylogenetic investigation of DENV-2 isolates from 2017 to 2021 originating from the northern states of Brazil. A total of 32 samples from DENV-2 isolates were analyzed, including 12 from Acre, 19 from Roraima, and one from Tocantins. Only one lineage of the Asian-American genotype and one lineage of the cosmopolitan genotype were observed: Lineage 1, Asian-American genotype (connection to Puerto Rico); Lineage 5, cosmopolitan genotype (connection to Peru). Our results provide important data regarding the study of DENV genotypes and lineage distribution and open up possibilities for probable introduction and dissemination routes.

## 1. Introduction

Dengue is an arboviral disease caused by the dengue virus (DENV–*Orthoflavivirus denguei*) [1]. DENV belongs to the *Flaviviridae* family, the *Orthoflavivirus* genus, in which member species possess a single-stranded positive-sense RNA genome and a viral envelope. Virus transmission occurs primarily through *Aedes* mosquito bites [2]. DENV exhibits four antigenically distinct serotypes (DENV-1, DENV-2, DENV-3, and DENV-4), and each serotype is further divided into several genotypes [3]. DENV infection can lead to a wide range of symptoms, from asymptomatic to severe [4].

DENV is spreading at an alarming rate worldwide, making it the fastest-expanding arbovirus [5]. Over the past few decades, the incidence of DENV has increased in tropical and subtropical countries, transforming DENV into an urgent global public health problem [5]. In addition to being the most prevalent arbovirus, DENV is also the most widely disseminated and is endemic in nearly all inhabited regions of the world. DENV is responsible for an estimated 100 million symptomatic clinical cases of infection per year, 500,000 cases of severe dengue, and approximately 22,000 deaths [6]. According to a World Health Organization (WHO) report [6], between Epidemiological Week (EW) 1 and EW 52 of 2022, a total of 2,809,818 dengue cases were reported, resulting in an accumulated incidence rate of 282.96 cases per 100,000 population. As of EW 1-35 of 2023, dengue remains the most predominant arbovirus, accounting for 75% of cases (1,530,940) [6,7,8].

Serotypes DENV-1 and DENV-2 have been widely circulating in South America. DENV-2, in particular, has contributed significantly to dengue-related mortality in countries such as Brazil [7,8]. The cocirculation of multiple serotypes can increase the risk of serious infections due to the risk of coming into contact with more than one serotype [9]. The genetic information presented in various studies of the evolution and global spread of DENV-2 genotypes highlights the importance of performing additional research into phenotypic variations, host adaptations, and the survival of its strains, which influence the epidemiology of DENV-2 and promote its spread in endemic areas [9,10,11]. In this context, the introduction of different serotypes and their ability to persist in the environment over time may be related to the unpredictability of clinical cases of reinfection with different serotypes [9]. In addition, the capacity for viral dissemination may be associated with more virulent strains of DENV-2 [9,10].

Among the four serotypes, DENV-2 is the most common cause of dengue epidemics on a global scale and, particularly in South American countries, is recognized for its association with cases of severe disease [11,12]. DENV-2 genotypes are constantly evolving due to their high mutation rate and rapid migration, which may lead to new lineages. The simultaneous circulation of different strains of DENV-2 in the country may be associated with a greater number of epidemic outbreaks and severe manifestations of the disease when compared to the other three dengue serotypes. The emergence and cocirculation of new DENV-2 lineages, replacing earlier lineages, have been previously reported [13,14,15,16,17,18].

There are five DENV-2 genotypes identified by their region of origin: Caribbean and South Pacific (genotype I or American); Taiwan, Philippines, New Guinea prototype virus, and the 1964 Thailand strain (genotype II or cosmopolitan); strains from Vietnam, Jamaica, and Thailand (genotype III or Asian-American); Indonesia, Seychelles, Burkina Faso, and Sri Lanka (genotype IV or Asian I and II); and rural areas of Africa (genotype V or sylvatic) [19,20,21,22].

The extensive coastline of the northern region of Brazil and its border with various countries, such as Bolivia, Peru, Colombia, Venezuela, Guyana, Suriname, and French Guiana, can significantly facilitate the introduction of diseases and frequent outbreaks [23,24,25]. Monitoring the continuous spread of arboviruses worldwide, understanding the likely causes of reemergence in regions where the virus previously circulated, and conducting epidemiological surveillance in endemic regions are essential for prevention and control measures to reduce vector proliferation and minimize the risk of infection [22,26].

Complete viral genome analysis allows the identification of DENV serotypes and genotypes, which is important for understanding the circulation of the virus, especially in endemic areas. This approach can help to determine the origin of isolated viruses, their introduction and dissemination routes within a region, and the identification of geographical provenance.

In this study, we conducted a phylogenetic investigation of DENV-2 in the northern region of Brazil, which contributes to the understanding of circulating genotypes and lineages and elucidates the influence of neighboring countries on strain diversity and subsequent lineage replacement. The obtained information provides essential insights into DENV-2 genotypes and lineage distribution, as well as identifying probable introduction routes.

## 2. Materials and Methods

### 2.1. Study

This is an observational, descriptive, and retrospective study. The identification and processing of samples, laboratory techniques, and data registration and analysis procedures were conducted at the Department of Arbovirology and Hemorrhagic Fevers of the Evandro Chagas Institute. Experimental analysis procedures were conducted according to the norms and criteria required by the Internal Biosafety Committee. This study did not involve the use of animals, humans, or the environment as direct research subjects, thus excluding the need for submission to the research ethics committee. Only isolated samples of DENV deposited and belonging to the institutional biorepository were used. Thus, DENV-2 samples from Northern Brazilian States (Acre, Tocantins, and Rondônia States) collected between 2017 and 2021 were eligible.

### 2.2. Viral Samples

The isolates correspond to DENV-2 strains from serum samples obtained through surveillance, and the strains were serotyped and confirmed as DENV-2 prior to our analysis. We retested using RT–qPCR for serotype 2 confirmation and sequenced our samples. The samples are derived from the epidemiological surveillance of arboviruses at the Evandro Chagas Institute. All biological samples were confirmed by molecular biology testing, subsequently isolated in cell cultures, and stored in liquid nitrogen until use. Prior to analysis, the samples were registered and presented based on the following parameters: (I) place of origin/federal unit (FU); identification (ID); (II) collection date (CD); (III) serotype; (IV) sample type; and (V) cycle threshold (CT) value (Table S1).

### 2.3. RNA Extraction and Purification

For sample extraction, 140 µL of cell culture supernatant was used with the QIAamp^®^ Viral RNA Mini Kit (Qiagen, Hilden, Germany) following the manufacturer’s recommendations. Total RNA was quantified using the Qubit^®^ 2.0 Fluorometer (Thermo Fisher Scientific, Waltham, MA, USA) with the Qubit^®^ RNA HS Assay kit and the Bioanalyzer 2100 (Agilent Technologies, Santa Clara, CA, USA) with the Agilent RNA 6000 Pico kit following the respective manufacturer’s protocols. Subsequently, the samples were stored in a freezer at −70 °C until further use.

### 2.4. RT–qPCR and Viral Isolation

All samples were pretested for viral isolation confirmation using molecular biology techniques for DENV serotype 2. The assay was performed using the SuperScript III Platinum One-Step Quantitative RT–PCR System Rox Kit (Thermo Fisher Scientific, Waltham, MA, USA), which included a set of oligonucleotide primers and dual-labeled fluorescent probes (Taqman, Thermo Fisher Scientific, Waltham, MA, USA) for in vitro qualitative detection of DENV-1–4. The 25 µL reaction consisted of 12.5 µL of a 2X SuperScript III Platinum RT–PCR master mix, 2.2 µL of nuclease-free water, 0.45 µL of each Taqman probe (final concentration 180 nM), 0.25 µL of the D1-D4 probe, 5 µL of extracted RNA, and forward and reverse primers as follows: 1.0 µL D1 (D1, F-5′, CAAAAGGAAGTCGYGCAATA, 3′; R-5′, CTGATGAATTCTCTCTGCTRAAC, 3′, final concentration 1 µM), 0.5 µL D2 (D2, F-5′, CAGGCTATGGCACYGTCACGAT, 3′; R-5′, CCATYTGCAGCARCACCATCTC, 3′, final concentration 500 nM), 1.0 µL D3 (D3, F-5′, GGACTRGACACACGCACCCA, 3′; R-5′, CATGTCTCTACCTTCTCGACTTGYCT, 3′, final concentration 1 µM), and 0.5 µL D4 (D4, F-5′, TTGTCCTAATGATGCTRGTCG, 3′; R-5′, TCCACCYGAGACTCCTTCCA, 3′, final concentration 500 nM).

In a 7500 Fast Real-Time PCR system (Thermo Fisher Scientific, Waltham, MA, USA), the RT–qPCR assays were performed under the following cycling conditions: an initial RT step at 50 °C for 30 min, a denaturation step at 95 °C for 2 min, 45 cycles of 15 s at 95 °C, and a final extension step at 60 °C for 1 min. The samples were analyzed in duplicate and confirmed as positive if the average CT value was below 37. All DENV-2 samples were selected for viral isolation and genomic analysis of the supernatants. The assay was validated using positive controls (DENV-1–4) and negative controls (nuclease-free water) [27].

For viral isolation in cell culture and the propagation and maintenance of cells, L-15 medium was utilized. The cells were kept at approximately 28 °C and monitored daily until the 7th day post-infection (DPI). Confluent monolayers were passaged in sterile 16 × 125 mm cell culture tubes and maintained with a cell culture area of 20 cm^2^ in a TPP brand or a similar 25 cm^2^ brand dish. On the seventh day after inoculation, aliquots of the infected cell suspension were collected for the indirect immunofluorescence test (IFI). Subsequently, the sample was stored at −70 °C until use.

A cell line derived from *Aedes albopictus*, clone C6/36 (ATCC: CRL1 660), was used. The cells were seeded in 10 mL culture tubes containing 1.5 mL of modified Leibowitz medium with glutamine (L-15) supplemented with 2.95% triptose phosphate, nonessential amino acids, antibiotics (penicillin and streptomycin), and 2% fetal bovine serum. Cells were inoculated at a 1:10 ratio of serum to modified L-15 and observed daily for a period of 7 days or until a cytopathic effect (CPE) was observed. Upon completion, the isolates were frozen until further processing for cDNA synthesis and genomic library preparation [27].

### 2.5. Synthesis of cDNA Double Strands and the Genomic Library

The cDNA preparation from RNA for genomic sequencing started with the synthesis of the first and second cDNA strands using the SuperScriptTM VILO^TM^ MasterMix kit (Thermo Fisher Scientific, Waltham, MA, USA) and NEBNext^®^ Second Strand Synthesis Module (New England BioLabs, Ipswich, MA, USA), respectively. The cDNA purification reaction was performed using the PureLink^®^ PCR Purification Kit (Thermo Fisher Scientific, Waltham, MA, USA). All steps followed the manufacturer’s recommendations for the respective kits. The genomic library was prepared following the instructions of the Nextera XT DNA kit (Illumina, San Diego, CA, USA). Quantification and fragmentation level assessment were performed using the DNA HS Kit Assay on the Qubit 4.0 instrument and the Agilent RNA 6000 Pico kit in the Bioanalyzer 2100 instrument, respectively. Upon confirming that the library had the desired quantity and size of fragments, sequencing was performed on the NextSeq 500 platform (Illumina) using the NextSeq 500/550 High Output Kit v2.5 (300 cycles) and paired-end methodology, as recommended by the manufacturer.

### 2.6. Bioinformatics and Phylogenetic Analysis

The data generated by NextSeq (Illumina) in .bcl format was converted to .fastq format using the bcl2fastq2 program [28], resulting in two files, R1 and R2. These files served as input for the initial data processing program. The generated data underwent an initial step of preprocessing the raw sequences, including adapter removal, filtering out low-quality reads (below Q20), and removing ambiguous bases using the Fastp v0.23.1 program [29]. The reads obtained from the previous step were assembled using the reference mapping methodology with the Bowtie2 v2.5.1 program [30]. These reads were mapped to the reference genome of DENV-2 (NC_001474) (available in the National Center for Biotechnology Information, NCBI, RefSeq database) using the Fastp v0.23.1 program to obtain consensus sequences. Geneious v.9.1.8 software [31], SAMtools v1.16.1 [32], and Bamtools v2.5.2 [33] software were used for the analysis and extraction of relevant information through manipulation and analysis of SAM and BAM files, including the extraction of read coverage information.

The sequences obtained from the complete genome of DENV-2 were aligned with sequences, including complete and partial genomes, from different genotypes of DENV-2 available in GenBank using MAFFT v.7 software [34]. Before constructing the phylogenetic tree, phylogenetic signal verification were performed to ensure the reliability of the analyzed data. TREE-PUZZLE v. 5.3 was used for this purpose, with the input file being the alignment of the sequence set in.phylip format (generated by Geneious v.9.1.8). The program employs the maximum likelihood methodology (ML) and produces a triangular diagram image for analysis [35].

After positive phylogenetic signal confirmation, the phylogenetic tree was built using ML [36] phylogenetic inference with a fixed bootstrap value of 1000 replicates using IQ-TREE v.2 [37,38]. The best nucleotide substitution model was selected according to ModelFinder incorporated into the IQ-TREE program v.2 [37,39]. The phylogenetic tree was rooted at the midpoint [40]. Tree visualization and editing were performed using FigTree v.1.4.4 [41] and Inkscape v.1.1 [28] software.

## 3. Results

A total of 32 serum samples were tested and confirmed for DENV-2, including 12 from Acre, 19 from Roraima, and one from Tocantins. The confirmed samples were isolated in cell cultures. The obtained isolates were analyzed by sequencing. All samples confirmed for DENV-2 were successfully isolated. The full genome sequences obtained were aligned through reference mapping with the reference genome of DENV-2 (NC_001474). A set of 124 complete and partial DENV-2 genome sequences available in GenBank, representing the genotypes circulating around the world, was aligned with this study sequences. The sequenced genomes were identified by the genotypic clade into which they were grouped. All sequences included in the phylogenetic inference are listed in the Supplementary Materials (Table S2). Before constructing the phylogenetic tree, the phylogenetic signals were checked to attest to the quality and, therefore, reliability of the data analyzed. The highly resolved tree coordinates (consistent) concentrate at the tips of the triangle, equivalent to 97.4%; the partially resolved topology is represented in the side rectangles, accounting for 1.2%; and the unresolved tree topology is centered, amounting to 1.4% (Figure S1). Since the proportion of unresolved tree topology is below 30%, a positive phylogenetic signal are identified, indicating the high quality of the analyzed data.

**Figure 1:**
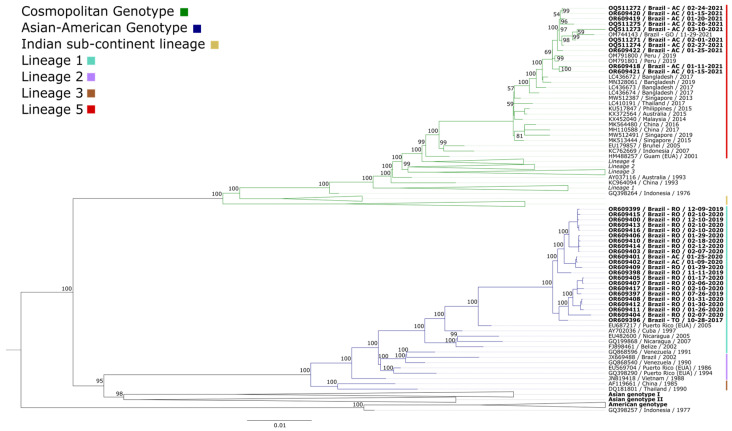
Maximum likelihood phylogenetic tree based on the alignment of the dataset comprising 156 DENV-2 genome sequences. The scale bar indicates the evolutionary distance in the number of nucleotide substitutions per site, and the bootstrap values are indicated on the main branches, with a focus on the cosmopolitan genotype (represented in green) and the Asian-American genotype (blue). Lineage 5 (cosmopolitan genotype) is represented in red. Lineage 1 (Asian-American genotype) is represented in cyan. The cosmopolitan genotype is divided into lineages 1–6. The Asian-American genotype is divided into lineages 1–4. The strains of the other genotypes not found are not described in the figure. The unclassified cosmopolitan genotype sequences do not belong to any of the six lineages. The name and information of the isolates characterized in this study are highlighted, including the sample identification number, country, state of origin, and collection date.

Based on the obtained alignments, phylogenetic analysis was performed. The phylogenetic tree was constructed using maximum likelihood, with the GTR+F+I+G4 nucleotide substitution model identified as the best fit. For comparison purposes, 124 sequences were included. The obtained sequences were grouped into a monophyletic clade belonging to the cosmopolitan genotype and a paraphyletic clade belonging to the Asian-American genotype (Figure 1). The names of the isolates used for comparison include the GenBank accession number, country of origin, and year of isolation.

The phylogenetic analysis of DENV-2 demonstrated the circulation of the Asian-American and cosmopolitan genotypes within the northern region (Acre, Rondônia, and Tocantins). The choice of the comparison dataset, although restrictive in terms of global diversity, enabled a more in-depth and detailed investigation of the phylogenetic relationships within the local context, providing valuable insights into the evolution and spread of lineages in the region under study.

## 4. Discussion

The emergence of arboviruses in previously unaffected areas poses significant challenges to public health [42,43,44]. Genomic surveillance plays a crucial role in monitoring the introduction of emerging infectious diseases and analyzing circulating viruses [45,46,47]. Therefore, efficient sequencing protocols and subsequent data sharing are essential for sequence comparison [46,47]. This approach enables the acquisition of information about viral introduction and dispersion routes, evolutionary implications, transmission pathways, and the design of effective epidemic containment measures [44,45,46,47,48].

Dengue is endemic in all regions of Brazil, and frequent outbreaks represent a significant public health problem. Several dengue serotypes and genotype changes have been observed in recent decades [15,16,17,19,20]. More recently, the reemergence of serotype 3 has been reported in the northern region of Brazil, associated with the emergence of a new lineage of genotype III (Indian subcontinent) in the Americas [18]. Because dengue is also endemic in neighboring countries such as Brazil, there is a persistent risk of virus importation and transmission of new serotypes and an increased likelihood of genetic divergence [9]. This highlights the need for early detection, surveillance, and continuous monitoring of DENV dissemination in Brazil and neighboring countries [17,18].

The Asian-American genotype of DENV-2 was introduced to the Americas from the Asian continent in the early 1980s and spread widely throughout the continent [49]. From the 1990s onward, the virus was reintroduced to Brazil through strains originating from the Caribbean [49,50,51]. Reports describe the Caribbean as the primary source of all DENV-2 lineages that were subsequently disseminated to continental regions, while Brazil, Venezuela, and Nicaragua were identified as major secondary centers of DENV-2 dissemination to other countries within South America [49]. Therefore, the phylogeographic pattern of DENV-2 in the Americas can be attributed to a model of short-distance transmission [49]. In this model, the virus originated from a central point in the Caribbean and spread to nearby continental regions, which act as secondary centers of dissemination, transmitting the virus to neighboring countries on the continent [52].

**Figure 2:**
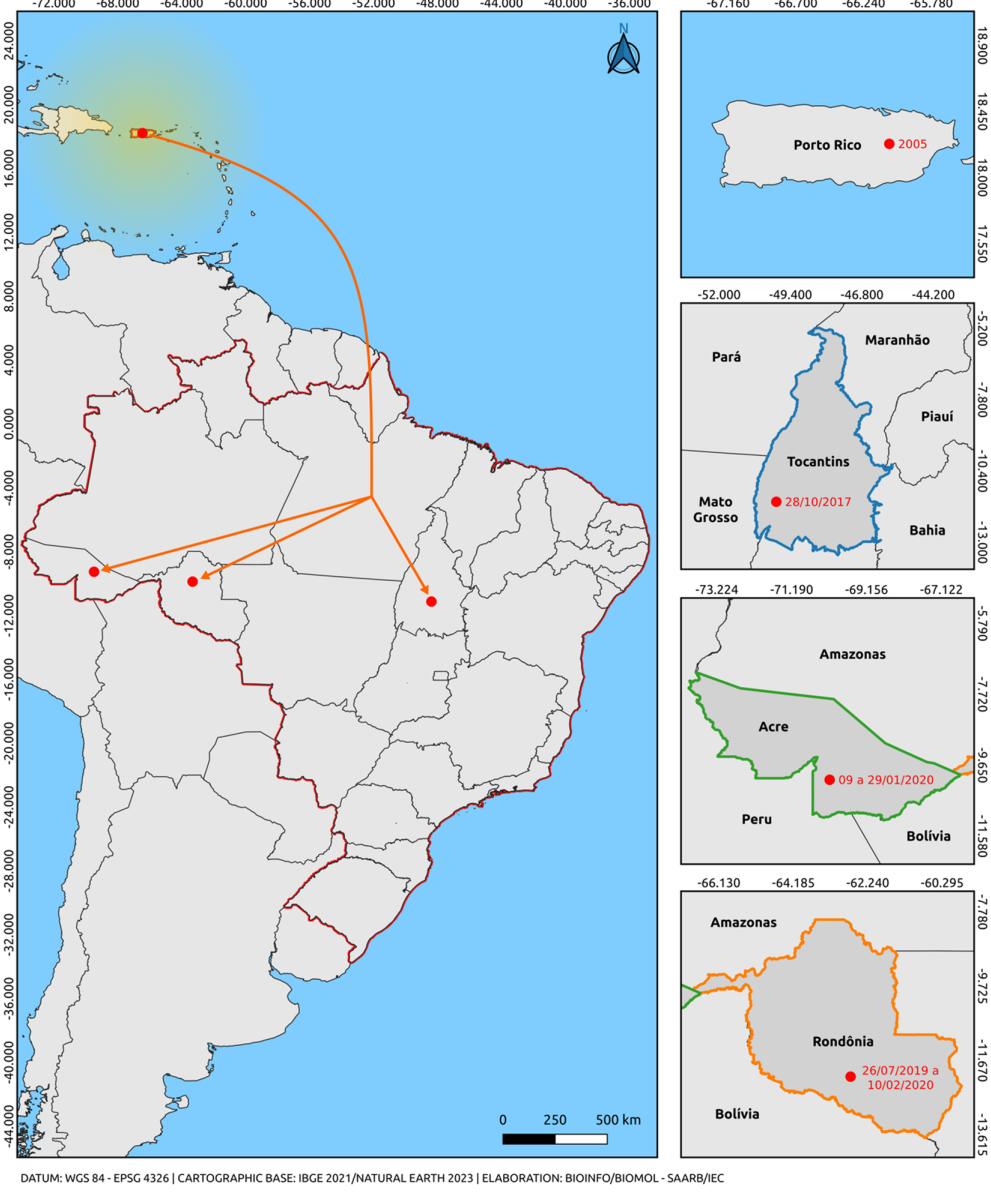
Map illustrating the possible route of introduction and dispersal of the Asian-American genotype of DENV-2 suggested by this study. The orange arrow indicates parallel dispersion from Puerto Rico through an unmapped route (Puerto Rico-Brazil/Tocantins, Acre, and Rondônia). The bifurcation region indicates only the intersection corresponding to parallel dispersion for the states of Acre, Tocantins, and Rondônia, not that the dispersion route necessarily includes the state in which the bifurcation region is located. In other words, the strains recorded in Brazilian states prior to 2020 likely originated from Puerto Rico. The colors delineate the states and the intersections between the regions of the countries (Blue: Tocantins, Brazil; Green: Acre, Brazil; Orange: Rondônia, Brazil; Red: border region with Brazilian states). The yellow gradient circle indicates potential strain dispersion routes to regions in the Americas and neighboring countries, originating from Puerto Rico as a secondary dissemination center. The map was created using QGIS software v.3.28, available at https://qgis.org/pt_BR/site/ (accessed on 9 June 2023).

Only one lineage of the Asian-American genotype was observed. All sequences of the Asian-American genotype used in this study segregate into a paraphyletic subgroup aligned with sequences from an American clade (Puerto Rico, Cuba, Nicaragua, Belize, Venezuela, and Brazil), well supported by high bootstrap values. The sequences of the Asian-American genotype lineage 1 positioned themselves in the American clade, closer to the basal strain from Puerto Rico (EU687217), which behaves as more evolutionarily ancient compared with our sequences. These results suggest the predominant circulation of lineage 1, as already documented in the recent history of DENV-2. We highlighted in our study the circulation of the Asian-American genotype in the states of Tocantins, Acre, and Rondônia from 07/2019 to 02/2020 (Figure 2) [53,54]. These cases may suggest a geographical connection between the strain reported in Puerto Rico and parallel dispersion to the states of the northern region of Brazil, providing insights into the most likely dispersion route of this genotype in Brazil.

The most significant finding of this study is the identification of sequences stemming from an outbreak that occurred in the state of Acre, Brazil, which phylogenetically clustered within the cosmopolitan genotype clade. This is noteworthy considering that previous studies have only documented the circulation of the Asian-American genotype in Brazil [52,53,54]. The strain reported in Goiás represents the second official record of the circulation of this genotype in the Americas, following an outbreak that occurred in Madre de Dios, Peru, in 2019. Madre de Dios is an extensive territorial area that borders the Amazon Region [45,46,47]. Recent reports have highlighted the widespread dissemination of the cosmopolitan genotype of DENV-2 in the Brazilian territory. These reports indicate that, after several entries into the country, this genotype has already been established in all geographic regions of Brazil [55,56].

In the late 1990s, in the Yucatán Peninsula, Mexico, the first case of a cosmopolitan strain of the DENV-2 virus in the Americas was documented. However, only one viral strain was isolated during this period and was the subject of study [45]. This specific genotype is present in various parts of the world, replacing variants that were previously prevalent in several Asian regions [45]. Based on recent information, it is possible to suggest a significant introduction of the cosmopolitan genotype of DENV-2 from Asian regions to Madre de Dios, Peru. This introduction appears to have originated from strains previously recorded in Dhaka, Bangladesh. The cosmopolitan genotype has recently been identified in Madre de Dios and is spreading globally, indicating broad dissemination of its lineages [45,46].

Our findings reinforce what we proposed in our partial report regarding the introduction of a new genotype, raise awareness about its dissemination, and provide insights into the cocirculation of multiple lineages in the Americas [56]. Although a phylogenetic relationship was observed between the only case of the cosmopolitan genotype in the Midwest region of Brazil and the cases described in Madre de Dios, Peru [45,46], no geographical linkage has been established between these regions that do not share borders [46,47,48].

**Figure 3:**
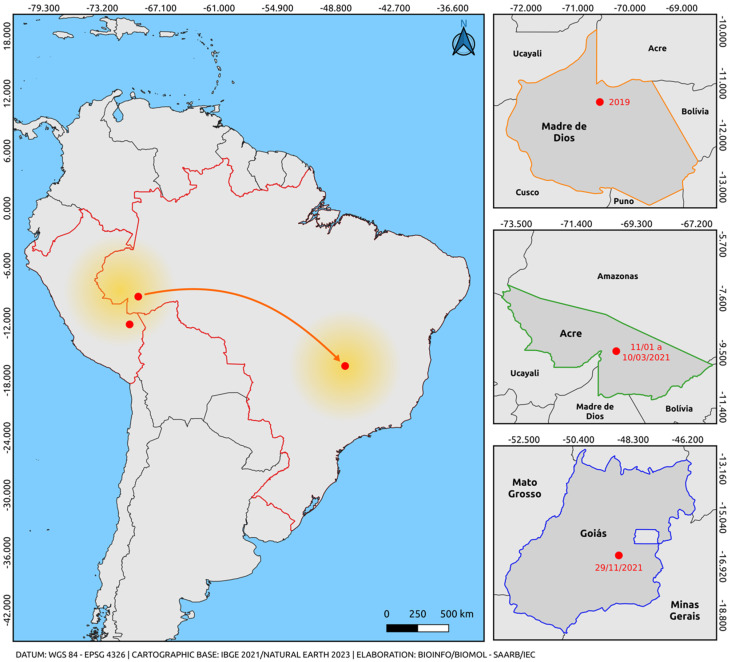
Map illustrating the possible route of introduction and dispersal of the DENV-2 cosmopolitan genotype. The orange arrow indicates only the departure from the point of origin and arrival through an unmapped route (Madre de Dios, Peru) (Acre/Brazil-Goiás/Brazil), based on the analysis of sequences obtained from reports in the last five years and the sequences from this study. The highlighted arrow is not related to the virus dispersion route; it only indicates the departure from the point of origin (Madre de Dios, Peru, and Acre, Brazil) and the destination (via an unmapped route) of Goiás, Brazil. The colors represent the states and intersections between regions of the countries (Orange: Madre de Dios, Peru; Green: Acre, Brazil; Blue: Goiás, Brazil; Red: border region between Brazil and Peru). The yellow gradient circle indicates the potential spread of the strain to other regions of Brazil and neighboring countries. The map was created using QGIS software v.3.28, available at https://qgis.org/pt_BR/site/ (accessed on 9 June 2023).

Ten sequences obtained in this study clustered within a well-supported South American clade, supported by high bootstrap values, including two Peruvian sequences and the only sequence previously described in Brazil. Our previous report revealed the first evidence of the cosmopolitan genotype in the northern region of Brazil during a DENV-2 outbreak in the state of Acre, northern region, in early 2021, months before the single detection case in Goiás [46,56]. Samples in this study were collected between January and March 2021, demonstrating the circulation of this genotype prior to the reported case in Goiás. Phylogenetic inference also revealed that the South American clade was positioned among sequences belonging to lineage 5 of the genotype. These cases establish a closer geographic connection between findings in Peru and the central-west region of Brazil, providing insights into the most likely route of introduction of this genotype into Brazil from the border with Peru in the Madre de Dios Region (Figure 3).

Although the persistence and local evolution of some strains are possible, the crucial element for the dynamics of DENV-2 epidemics in the state seems to have been the introduction of Peruvian strains (OM791800; OM791801). The cosmopolitan genotype of DENV-2 is currently in circulation throughout Brazil, as described in previous research [46,55,56]. A recent study identified multiple introductions of this genotype in Brazil originating from Peru, resulting in several clusters that have spread throughout the country with different levels of dissemination. These results emphasize the need to monitor the introduction of new genotypes/lineages in a specific region. Rapid changes in these genotypes or lineages can occur and could be attributed to fitness advantages, resulting in a greater ability to reach higher levels of viremia in humans. This leads to an increased rate of transmission between humans and mosquitoes, which is also associated with the circulation of different genotypes in the same region.

We can infer that there was a large-scale introduction of the cosmopolitan genotype of DENV-2 in South America. Our data allow us to hypothesize that our strains, as well as those from Peru, may have derived from those reported in Dhaka, Bangladesh. However, further studies are needed to determine where its introduction occurred and how long it has been circulating. Considering the sample dates, we can infer that this genotype has been circulating in Brazil for at least two years, highlighting the importance of retrospective studies to investigate the dispersal pattern of cosmopolitan genotype lineages of DENV-2.

The circulation of different genotypes may have important implications for viral epidemic capacity. Considering the lack of retrospective studies evaluating the circulation period of different DENV-2 lineages, it is likely that multiple lineages coexist in the same region. To understand the dispersion route of both detected lineages from the point of origin, more information on sample collection and sequencing between Puerto Rico and Brazil for the Asian-American genotype (line 1) and Bangladesh, Peru, and Brazil for the cosmopolitan genotype would be necessary. Consequently, a larger volume of data are needed to describe the evolutionary route.

The population sampled in our dataset represents a small fraction of DENV-2 cases since the first report of the cosmopolitan genotype in Brazil. This makes it impossible to define the introduction route and the time of virus circulation. To investigate the time of virus circulation in the region, retrospective studies with samples from other states are needed. However, our approach has proven robust for studying the spread of DENV-2 at a regional level, as long as the results are interpreted with caution within a solid biological framework. This study emphasizes the relevance of molecular research in the context of the origin and evolution of the dengue virus. The results point to the promising use of these studies as a tool capable of predicting epidemics in the context of genomic surveillance.

## 5. Conclusions

The DENV-2 serotype isolated from 2017 to 2021 in the northern region of Brazil, specifically the states of Acre, Roraima, and Tocantins, belongs to the Asian-American and cosmopolitan genotypes. During this period, two distinct lineages of DENV-2 circulated: lineage 1 from the Asian-American genotype (with a geographic link to Puerto Rico) and lineage 5 from the cosmopolitan genotype (with a geographic link to Peru).

However, starting in 2021, the cosmopolitan genotype began to circulate in the state of Acre, with a reported strain in the state of Goiás in the same year. The discovery of the circulation of a new genotype in the Brazilian territory strengthens previous findings in South America regarding the circulation of the cosmopolitan genotype in the Americas. The occurrence of two distinct genotypes in the same region and in neighboring areas suggests the circulation of different genotypic lineages.

The dataset used in this study emphasizes the need for retrospective studies investigating the origin and introduction route of DENV-2, as well as possible nucleotide and amino acid alterations in the genome of isolates associated with mutations or particularities in the clinical manifestation of the disease or type of infection.

The detection of Asian-American and cosmopolitan genotypes alerts to the emergence of DENV-2 in neighboring regions with intense population exchange, where the inability to adhere to public policies for arbovirus control hinders the establishment of truly effective genomic surveillance.

## Data Availability

The sequences generated in this study were deposited in GenBank under the following accession numbers: OR609396-OQ511274.

## Supplementary Materials

The following supporting information can be downloaded at: https://www.mdpi.com/article/10.3390/tropicalmed9010017/s1; Table S1. List of selected samples in the study; Table S2. List of sequences included in phylogenetic inference; Figure S1. Representation of the positive phylogenetic signal for the set of studied sequences.
