# Supplementary material for: Detection of a Multiple Circulation Event of Dengue Virus 2 Strains in the Northern Region of Brazil
Source: Trop Med Infect Dis. 2024 Jan 9;9(1):17. doi: 10.3390/tropicalmed9010017 (PMC10818346; doi:10.3390/tropicalmed9010017)
Supplement: Supplementary file 1 [file tropicalmed-09-00017-s001.zip › tropicalmed-2734318-supplementary.pdf]

Tropical Medicine and Infectious Disease

MDPI

Detection of a Multiple Circulation Event of Dengue Virus 2 Strains in the Northern Region of Brazil

Supplementary Materials:

Table S1. List of selected samples in the study.

#	FU	ID	CD	Serotype	Sample	CT Value
1	TO	OR609396	28/10/2017	Dengue 2	Serum	14,3
2	RO	OR609397	26/07/2019	Dengue 2	Serum	21,4
3	RO	OR609398	11/11/2019	Dengue 2	Serum	21,6
4	RO	OR609399	09/12/2019	Dengue 2	Serum	22,1
5	RO	OR609400	10/12/2019	Dengue 2	Serum	23,4
6	RO	OR609411	26/01/2020	Dengue 2	Serum	20,7
7	RO	OR609404	07/02/2020	Dengue 2	Serum	22,7
8	RO	OR609405	17/01/2020	Dengue 2	Serum	22,1
9	RO	OR609406	29/01/2020	Dengue 2	Serum	29,4
10	RO	OR609407	06/02/2020	Dengue 2	Serum	24,07
11	RO	OR609408	31/01/2020	Dengue 2	Serum	24,5
12	RO	OR609412	30/01/2020	Dengue 2	Serum	21,6
13	RO	OR609403	07/02/2020	Dengue 2	Serum	21,7
14	RO	OR609410	18/02/2020	Dengue 2	Serum	23,7
15	RO	OR609413	10/02/2020	Dengue 2	Serum	25,7
16	RO	OR609414	12/02/2020	Dengue 2	Serum	19,7
17	RO	OR609415	10/02/2020	Dengue 2	Serum	20,3
18	RO	OR609417	10/02/2020	Dengue 2	Serum	20,6
19	RO	OR609409	29/01/2020	Dengue 2	Serum	25,3
20	RO	OR609416	07/01/2020	Dengue 2	Serum	22,1
21	AC	OR609401	25/01/2020	Dengue 2	Serum	20,2
22	AC	OR609402	09/01/2020	Dengue 2	Serum	Unrealized
23	AC	OR609421	15/01/2021	Dengue 2	Serum	15,7
24	AC	OR609418	11/01/2021	Dengue 2	Serum	17,8
25	AC	OR609419	20/01/2021	Dengue 2	Serum	Unrealized
26	AC	OR609420	15/01/2021	Dengue 2	Serum	16,9
27	AC	OR609422	25/01/2021	Dengue 2	Serum	19,3
28	AC	OQ511272	24/02/2021	Dengue 2	Serum	19,9
29	AC	OQ511275	26/02/2021	Dengue 2	Serum	19,8
30	AC	OQ511273	10/03/2021	Dengue 2	Serum	17,9
31	AC	OQ511271	01/02/2021	Dengue 2	Serum	17,1
32	AC	OQ511274	27/02/2021	Dengue 2	Serum	15,5

Table S2. - List of sequences included in phylogenetic inference.

Access Number	Country	Collection Date	Genotype	Lineage
**OQ511273**	**Brazil**	**10-03-2021**	Cosmopolitan	5
**OQ511271**	**Brazil**	**01-02-2021**		
**OQ511274**	**Brazil**	**27-02-2021**		
**OQ511272**	**Brazil**	**24-02-2021**		
**OQ511275**	**Brazil**	**26-02-2021**		
**OR609420**	**Brazil**	**15-01-2021**		
**OR609419**	**Brazil**	**20-01-2021**		
**OR609422**	**Brazil**	**25-01-2021**		
**OR609418**	**Brazil**	**11-01-2021**		
**OR609421**	**Brazil**	**15-01-2021**		
OM744143	Brazil	29-11-2021		

Access Number	Country	Collection Date	Genotype	Lineage
OM791800	Peru	2019		
OM791801	Peru	2019		
LC436672	Bangladesh	2017		
MN328061	Bangladesh	2019		
LC436673	Bangladesh	2017		
LC436674	Bangladesh	2017		
MW512387	Singapore	2013		
LC410191	Thailand	2017		
KU517847	Philippines	2015		
KX372564	Australia	2015		
KX452040	Malaysia	2014		
MK564480	China	2016		
MH110588	China	2017		
MW512491	Singapore	2019		
MK513444	Singapore	2015		
EU179857	Brunei	2005		
KC762669	Indonesia	2007		
HM488257	Guam	2001		
KX380830	Singapore	2013		
KX380819	Singapore	2012		
KX380809	Singapore	2012		4
KY921904	Singapore	2014		
MW512379	Singapore	2012		
KX380836	Singapore	2013		
KU517846	Indonesia	2014		
MW512466	Singapore	2016		
AY858036	Indonesia	2004		
MK578532	China	2016		
MW512354	Singapore	2009		
KM279597	Singapore	2012		
JN851113	Singapore	2006		2
KM279604	Singapore	2008		
KU509272	Thailand	2009		
KC762660	Indonésia	2007		
KX452015	Malásia	2014		
KC762678	Indonésia	2010		
MW512414	Singapore	2014		
KU517845	Papua New Guinea	2013		
MH985858	Australia	2016		
KC762658	Indonesia	2007		
EU081177	Singapore	2005		
EU081180	Singapore	2005		
JN851124	Singapore	2005		-
KC762672	Indonesia	2008		-
KC762680	Indonesia	2010		-
JX470186	China	2010		3
EU482640	Vietnam	2006		
EU179859	Brunei	2006		
MW512376	Singapore	2012		
KT187557	China	2014		
MG189962	Tanzania	2014		
AY858035	Indonesia	2004		

Access Number	Country	Collection Date	Genotype	Lineage
KU365902	Taiwan	2015		
MW512450	Singapore	2016		
MF004385	France	2014		
AY037116	Australia	1993		
KC964094	China	1993		
EF051521	China	2001		-
KU509274	Philippines	2010		-
KF744397	Philippines	2001		1
MW512489	Singapore	2018		
DQ645553	Taiwan	2002		
DQ645554	Taiwan	2002		
KF744398	Filipinas	2005		
JN851131	Singapore	2005		
MW512409	Singapore	2013		
AB189122	Indonesia	1998		
GQ398264	Indonesia	1976		
EU056810	Burkina Faso	1983		
GU131843	Burkina Faso	1986		-
GQ398262	Indonesia	1976		-
KJ830750	Saudi Arabia	2014		-
KJ010186	Pakistan	2013		-
GQ252677	Sri Lanka	2003		Indian Sub-continent
MF156247	China	2015		
MH110592	China	2017		
MH822948	India	2012		
JQ955623	India	2009		
MW512372	Singapore	2012		
MW512490	Singapore	2019		
MG779194	Kenya	2017		
KY672948	China	2013		
KY849763	Laos	2010		
DQ181797	Thailand	2001	Asian I	-
GU131897	Camboja	2007		-
FM210211	Vietnam	2003		-
DQ181798	Thailand	1999		-
GQ868545	Thailand	1996		-
DQ181802	Thailand	1988		-
DQ181805	Thailand	1979		-
AJ487271	Thailand	1974		-
NC_001474	Thailand	1964		-
HQ891023	Taiwan	2008		-
HQ891024	Taiwan	2008		-
AF204177	China	1989	Asian II	-
AF038403	Papua New Guinea	1944		-
FJ906959	Papua New Guinea	2008		-
KF704358	Cuba	1981		-
KF744400	Philippines	2000		-
KF744407	Philippines	1996		-
KJ734727	Taiwan	2014		-
EU482600	Nicaragua	2005		-
**OR609399**	**Brazil**	**09-12-2019**		-
**OR609415**	**Brazil**	**10-02-2020**	Asian-American	-

Access Number	Country	Collection Date	Genotype	Lineage
**OR609400**	**Brazil**	**10-12-2019**		-
**OR609413**	**Brazil**	**10-02-2020**		-
**OR609416**	**Brazil**	**10-02-2020**		-
**OR609406**	**Brazil**	**29-01-2020**		-
**OR609410**	**Brazil**	**18-02-2020**		-
**OR609414**	**Brazil**	**12-02-2020**		-
**OR609403**	**Brazil**	**07-02-2020**		-
**OR609401**	**Brazil**	**25-01-2020**		-
**OR609402**	**Brazil**	**09-01-2020**		-
**OR609409**	**Brazil**	**29-01-2020**		-
**OR609398**	**Brazil**	**11-11-2019**		-
**OR609405**	**Brazil**	**17-01-2020**		-
**OR609407**	**Brazil**	**06-02-2020**		-
**OR609417**	**Brazil**	**10-02-2020**		-
**OR609397**	**Brazil**	**26-07-2019**		-
**OR609408**	**Brazil**	**31-01-2020**		-
**OR609412**	**Brazil**	**30-01-2020**		-
**OR609411**	**Brazil**	**26-01-2020**		-
**OR609404**	**Brazil**	**07-02-2020**		-
**OR609396**	**Brazil**	**28-10-2017**		-
GQ199868	Nicaragua	2007		-
FJ898461	Belize	2002		-
AY702036	Cuba	1997		-
EU687217	Puerto Rico	2005		-
GQ868596	Venezuela	1991		-
JX669488	Brasil	2002		-
GQ868540	Venezuela	1990		-
EU569704	Puerto Rico	1986		-
GQ398290	Puerto Rico	1994		-
JN819418	Vietnam	1988		-
AF119661	China	1985		-
DQ181801	Thailand	1990		-
AF100469	México	1992		-
GQ868589	México	1983		-
EU056811	Peru	1995	American	-
GQ868592	Colombia	1986		-
HM582107	American Samoa	1972		-
HM582110	French Polynesia	1973		-
HM582117	Tonga	1974		-
EU056812	Puerto Rico	1977		-
GQ398257	Indonesia	1977		-

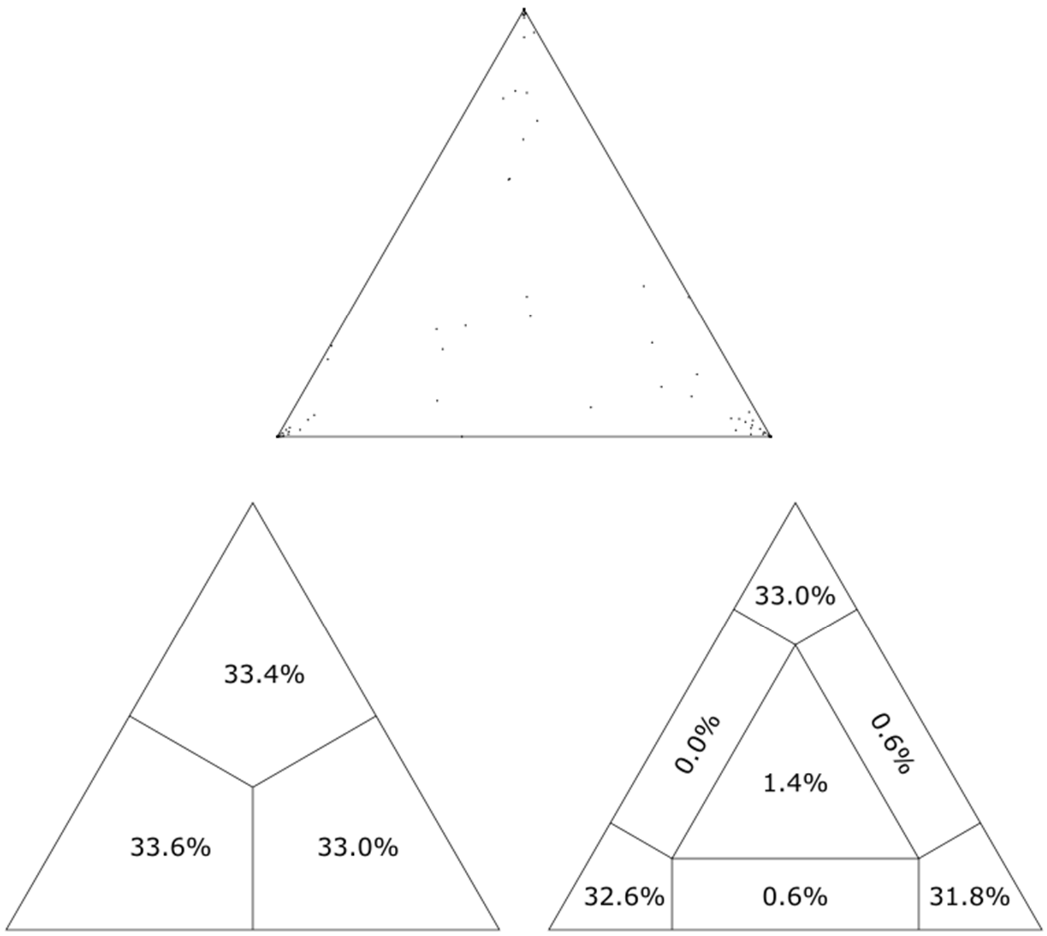

Figure S1. Representation of the positive phylogenetic signal for the set of studied sequences.
